# Machine learning application in soccer: a systematic review

**DOI:** 10.5114/biolsport.2023.112970

**Published:** 2022-03-16

**Authors:** Markel Rico-González, José Pino-Ortega, Amaia Méndez, Filipe Manuel Clemente, Arnold Baca

**Affiliations:** 1 Department of Didactics of Musical, Plastic and Corporal Expression, University of the Basque Country, UPV-EHU. Leioa, Spain; 2 BIOVETMED & SPORTSCI Research group. University of Murcia, San Javier. España; 3 Faculty of Sports Sciences. University of Murcia, San Javier. Spain; 4 Department of mechanics, design and industrial management, Faculty of engineering, University of Deusto, Bilbao, Spain; 5 Escola Superior Desporto e Lazer, Instituto Politécnico de Viana do Castelo, Rua Escola Industrial e Comercial de Nun’Álvares, 4900-347 Viana do Castelo, Portugal; 6 Instituto de Telecomunicações, Delegação da Covilhã, Lisboa 1049-001, Portugal; 7 Centre for Sport Science and University Sports, University of Vienna, Austria

**Keywords:** Team sports, Prediction, Algorithm, Computer science, Big data

## Abstract

Due to the chaotic nature of soccer, the predictive statistical models have become in a current challenge to decision-making based on scientific evidence. The aim of the present study was to systematically identify original studies that applied machine learning (ML) to soccer data, highlighting current possibilities in ML and future applications. A systematic review of PubMed, SPORTDiscus, and FECYT (Web of Sciences, CCC, DIIDW, KJD, MEDLINE, RSCI, and SCIELO) was performed according to the Preferred Reporting Items for Systematic Reviews and Meta-Analyses (PRISMA) guidelines. From the 145 studies initially identified, 32 were fully reviewed, and their outcome measures were extracted and analyzed. In summary, all articles were clustered into three groups: injury (n = 7); performance (n = 21), which was classified in match/league outcomes forecasting, physical/physiological forecasting, and technical/tactical forecasting; and the last group was about talent forecasting (n = 5). The development of technology, and subsequently the large amount of data available, has become ML in an important strategy to help team staff members in decision-making predicting dose-response relationship reducing the chaotic nature of this team sport. However, since ML models depend upon the amount of dataset, further studies should analyze the amount of data input needed make to a relevant predictive attempt which makes accurate predicting available.

## INTRODUCTION

Machine learning (ML) is the science that allows computers to act as humans and learn, improving their knowledge from data feed over time in an autonomous way in any area of life [1]; where sport is not an exception [2–4]. The use of ML allows coaches to continually gain new knowledge, continuously adding data which leads to a constant solution update. Therefore, as long as the most appropriate and constantly changing data sources are used, the sport scientist may predict the future indicating some pieces of advice. These highlights may provide information about training design to minimize the occurrence of injuries [4], inducing both players’ and teams’ performance improvements [5–7], and even predicting athletes with the most potential [8–10]. In fact, ML becomes more relevant in soccer due to its chaotic nature and the unpredictability of players’ behavior [11].

However, the use of a variety of algorithms that interactively learn from information becomes ML in a complex process in which the fundamental use of these algorithms allows the provision of more accurate data as long as new data is used [12, 13]. In brief, the algorithms used in ML are divided into (i) supervised learning (i.e., classification and regression) based on input/output data, and (ii) unsupervised learning (clustering) based on input data [14]. Some of the most considered ML predictor models are decision trees classifiers [3, 7], random forest [5, 7], and support vector machines [15, 16]. However, a wide range of other methods are being considered in soccer for injury, performance, and talent identification predictions [17, 18].

In practice, if data management is the main basis for producing the greatest levers in soccer, the use of predictive models has become the main challenge in the current era of big data. In this scenario, these models depend upon the quality and amount of dataset composed by attributes, also named as features or variables, which are the items of data that are used in ML. The new possibility to record and store data has allowed a deep training of learning models, being able to predict what will happen with a high degree of accuracy. For example, López-Valencia et al. [3] and Ayala et al. [2], through personal, psychological, and neuromuscular risk factors compared four different algorithms that were capable to predict the majority of injury incidences. This predicting may become crucial due to the high degree of economic loss that clubs experience when a player is injured [19]. However, this is not the only way to apply ML in soccer. Other authors have analyzed the use of different variables such as tactical, technical, psychological, and contextual variables to predict players’ and teams’ future performance [17, 20–22], or even to choose the players with the most potential [8–10].

So, due to the exponential increments that have arisen since the first ML models in soccer [23], the summarization and extraction of a set of conclusions remains crucial in order to identify and consider when to make decisions. To date, the publication of some narrative reviews has summarized the use of different predictive techniques in team sports (basketball, baseball, football, cricket, and soccer) [24] and the recent advances in the use of ML and statistical methods for the prediction of maximal oxygen uptake [25]. Beyond, to the best of authors knowledge, just two systematic reviews have summarized the use of ML. Cludino et al. [14] summarized the use of ML in general team sports (basketball, American football, Australian football, volleyball, soccer, and handball) for injury risk and performance analysis; while Herold et al. [11] focused their systematic review on the current applications and future directions of predicting models applied to the attacking game phase. However, no study has summarized the application of ML in soccer for all injury prevention, performance prediction, and talent identification. Therefore, the aim of the present study was to systematically identify original studies that applied ML with soccer data, highlighting current possibilities in ML and future applications. The present study may help team staff members in decision-making to predict dose-response relationships in order to reduce the chaotic nature of this team sport.

## MATERIALS AND METHODS

This systematic review was reported in accordance with the Preferred Reporting Items for Systematic Reviews and Meta-Analyses (PRISMA) guidelines [26–28] and guidelines for performing systematic reviews in sport sciences [29].

### Information sources

PubMed, SPORTDiscus, and FECYT (i.e., Web of Sciences, CCC, DIIDW, KJD, MEDLINE, RSCI, and SCIELO), were searched for relevant publications prior to February 19, 2021.

### Search strategy

Keywords and synonyms were entered in various combinations in the title, abstract or keywords: *(soccer OR football) AND (“deep learning” OR “machine learning”)*

Additionally, the reference lists of the studies retrieved were manually searched to identify potentially eligible studies not captured by the electronic searches. Finally, an external expert was contacted in order to verify the final list of references included in this scoping review in order to understand if there was any study that had not been detected through our research. Possible errata were searched for each included study.

### Eligibility criteria

The inclusion and exclusion criteria can be found in Table 1.

**TABLE 1:** Inclusion/exclusion criteria of selected studies.

Item	Inclusion Criteria	Exclusion Criteria
Population	Studies developed with soccer players	Studies developed with players from other team sports (basketball, rugby, Australian football, American football, futsal, etc.) or sport, even with videogames (i.e., FIFA computer games).
Intervention	The data was processed using machine-learning algorithms.	The data was not processed or not was processed using machine-learning methods.
Comparator	–	–
Outcome	Predictions were made about injury risk/prevention, performance, and/or talent identification.	Predictions about other issues (e.g., financial issues, social issues).
Study Design	The data were extracted during training sessions or matches.	The data were extracted in other moments that were unrelated to injuries, performance, and/or talent.
Additional criteria	Only original and full-text studies written in English	Written in languages other than English. Article types different from original (e.g., reviews, conference abstracts, etc.).

To locate potentially relevant studies, the screening of the title, abstract and reference list of each study was independently performed by the two authors (MRG and JPO). Additionally, they reviewed the full version of the included papers in detail to identify articles that met the selection criteria. An additional search within the reference lists of the included records was conducted to retrieve additional relevant studies. A discussion was conducted with a third author (AMZ) in cases of discrepancies regarding the selection process. Possible errata for the included articles were considered.

### Data Extraction

A data extraction was prepared in Microsoft Excel (Microsoft Corporation, Readmon, WA, USA) in accordance with the Cochrane Consumers and Communication Review Group’s data extraction template [30]. The Excel sheet was used to assess inclusion requirements and subsequently tested for all selected studies. The process was independently conducted by the two authors (MRG and JPO). Any disagreement regarding study eligibility was resolved in a discussion. Any full-text articles excluded, with reasons, were recorded. All records were stored in the datasheet.

### Data items

The following information was extracted from the included original articles: (1) participants (number of teams, number of matches, number of attempts); (2) data acquisition source/technology; (3) attributes, variables, or features; (4) aim of prediction; (5) ML issues (approach, algorithms, and prediction %); and concluding remarks and predicting.

### Methodological Assessment

A methodological assessment process was performed by the two authors (JPO and MRG) using an adapted version of the STROBE assessment criteria for cross-sectional studies [31], looking for studies eligible for inclusion. Each article was assessed based on 10 specific criteria: provide in the abstract an informative and balanced summary of what was done and what was found (Item 1); state specific objectives, including any prespecified hypotheses (Item 2); Give the eligibility criteria, and the sources and methods of selection of participants (Item 3); for each variable of interest, give sources of data and details of methods of assessment (measurement). Describe comparability of assessment methods if there is more than one group (Item 4); explain how quantitative variables were handled in the analyses. If applicable, describe which groupings were chosen and why (Item 5); give characteristics of study participants (Item 6); summarize key results with reference to study objectives (Item 7); discuss limitations of the study, considering sources of potential bias or imprecision. Discuss both direction and magnitude of any potential bias (Item 8); give a cautious overall interpretation of results considering objectives, limitations, multiplicity of analyses, results from similar studies, and other relevant evidence (Item 9); give the source of funding and the role of the funders for the present study and, if applicable, for the original study on which the present article is based (Item 10).

Any disagreement was discussed and solved by consensus decision. Each item was evaluated using numerical characterization (1 = completed; and, 0 = incomplete). As suggested by O’Relly et al. [31], each study rating was qualitatively interpreted according to the following law: the study has a risk of bias or low quality with a lower punctuation value than 7 points, while those studies with greater punctuation are considered as having a low risk of bias or as high quality.

## RESULTS

### Study identification and selection

**FIG. 1:**
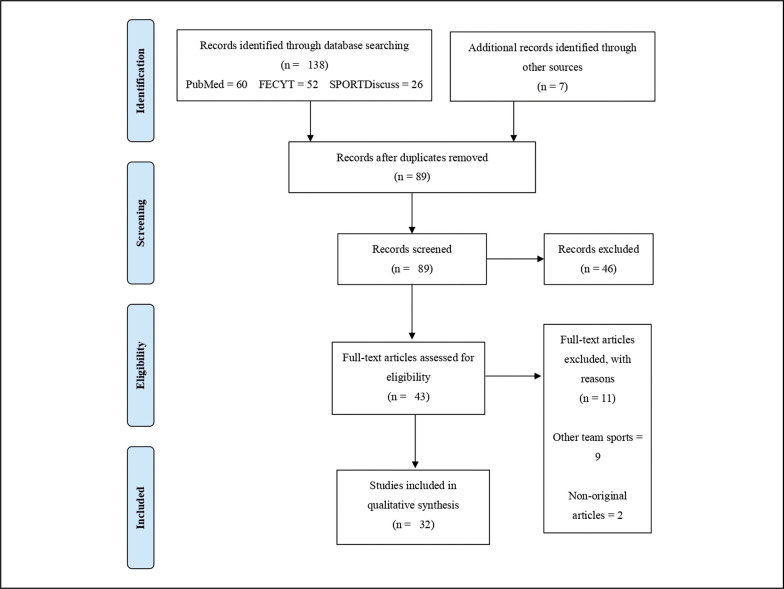
Flow diagram of the selection of studies.

The searching of databases identified a total of 138 titles (PubMed = 60; FECYT = 52; SPORTDiscus = 26). In addition, 7 studies were included from external sources. These studies were then exported to reference manager software (EndNote^TM^ X9, Clarivate Analytics, Philadelphia, PA, USA). Duplicates (56 references) were subsequently removed either automatically or manually. The remaining 89 articles were screened for their relevance based on titles and abstracts, resulting in the removal of a further 46 studies. Following the screening procedure, 43 articles were selected for in-depth reading and analysis. After reading full texts, a further 11 studies were excluded due to not meeting the eligibility criteria. Finally, 32 studies were included in the qualitative synthesis (Figure 1).

### Methodological quality

The overall methodological quality of the studies can be found in Table 2.

**TABLE 2:** Methodological assessment of the included studies.

Reference	1	2	3	4	5	6	7	8	9	10	Quality
Ayala et al. [2]	1	1	1	1	1	1	1	1	1	1	High
Barron et al. [8]	1	0	1	1	1	0	1	1	1	1	High
Bialkowski et al. [22]	1	0	0	1	1	0	1	1	0	1	Low
Bilek and Ulas [7]	1	0	1	1	1	1	1	0	1	0	Low
Bongiovanni et al. [48]	1	0	1	1	1	1	1	1	0	0	Low
Brooks et al. [20]	1	1	1	1	1	0	1	1	1	1	High
Campbell et al. [5]	1	0	0	1	1	0	1	1	1	0	Low
Chawla et al. [16]	1	0	1	1	1	0	1	1	1	0	Low
Ćwiklinski et al. [9]	1	0	0	1	1	0	1	0	1	1	Low
DiCesare et al. [18]	1	1	1	1	1	1	1	1	1	1	High
Dick and Brefeld [32]	1	0	1	1	1	0	1	0	1	1	Low
García-Aliaga et al. [33]	1	0	1	1	1	1	1	0	1	1	High
Geurkink et al. [34]	1	0	1	1	1	1	1	1	0	0	Low
Goes et al. [46]	1	1	1	1	1	0	1	1	1	1	High
Goes et al. [17]	1	1	1	1	1	0	1	1	0	1	High
Jaspers et al. [35]	1	0	1	1	1	1	1	1	1	1	High
Joseph et al. [23]	1	0	1	1	1	0	1	1	1	0	Low
Knauf et al. [36]	1	0	1	1	1	1	1	0	0	0	Low
Leser et al. [37]	1	0	1	1	1	1	1	1	1	1	High
Link and Hoering [38]	1	0	1	1	1	0	1	1	0	1	Low
López-Valenciano et al. [3]	1	0	1	1	1	1	1	1	1	1	High
Montoliu et al. [39]	1	0	1	1	1	0	1	1	0	0	Low
Oliver et al. [4]	1	0	1	1	1	1	1	0	1	1	High
Op De Beéck et al. [40]	1	0	1	1	1	1	1	1	1	0	High
Pappalardo and Cintia [6]	1	0	1	1	1	0	1	1	1	1	High
Perri et al. [21]	*Full text not found*										
Rodas et al. [15]	*Full text not found*										
Rommers et al. [41]	1	0	1	1	1	1	1	1	1	1	High
Rossi et al. [19]	1	0	1	1	1	1	1	0	0	1	Low
Sheng et al. [10]	1	0	0	1	1	0	1	0	1	0	Low
Szczepanski and McHale [42]	1	1	1	1	1	0	1	0	1	1	High
Zago et al. [43]	1	1	1	1	1	1	1	1	1	1	High

### Characteristics of individual studies

The characteristics of studies were extracted and clustered into three tables: ML and injuries (Table 3), ML and performance (Table 4), and ML and talent identification (Table 5).

**TABLE 3:** Stud ies that used ML to predict injuries in soccer.

Ref.	Participants	Data acquisition technology	Attributes/ Features/ Variables	Aim of prediction	Machine Learning			Practical application from predicting
					ML Approach	Algorithm	Accuracy	
Ayala et al. [2]	96 professionals from 4 teams (1^st^, 2^nd^). 18 injuries during 1 pre-season.	Surveys + Tools for biomechanical test	Personal risk factors (playing position, dominant leg, etc.) + Psychological risk factors (sleep diary and burnout) + Neuromuscular risk factors (postural, ROM, stability, flexibility)	Hamstring strain injuries (HIS)	Supervised	ADTree	> 78%	Sleep quality, history of HIS last season, hip flexion ROM (joint ranges of motion), and the angle of peak torque measured during eccentric (hamstrings) knee extension movements is important for predicting in-season HIS.

DiCesare et al. [18]	75 players from high-school academies (17 games)	Video recording + MEMS (accelerometer)	Head impacts	Brain change derived from these actions	-	Extreme Gradient Booster (XGBoost) based on trees classifier	> 83%	The number of head impacts can predict changes in the brain through longitudinal ML algorithms.

López-Valenciano et al. [3]	98 professional (1^st^ and 2^nd^) players 1 from 4 teams	Tools for biomechanical test (Y-balance device, dynamometer) + Personal (playing position, dominant leg, etc.) and psychological (sleep quality and burnout) surveys	Personal risk factors (playing position, dominant leg, etc.) + Psychological risk factors (sleep diary and burnout) + Neuromuscular risk factors (postural, ROM, stability, flexibility)	Lower extremity muscle injuries	Supervised	4 decision tree algorithms: C4.5 SimpleCart ADTree RandomTree	> 66%	The presented model could moderately help coaches in decision-making and training design to reduce the number and severity of muscle injuries.

Oliver et al. [4]	355 youths (from 10 to 18 years old) during 1 season.	Tools for biomechanical test	Injury incidence during 1 season + Pre-season screen: anthropometric, single leg CMJ and leg hop for distance, Y-balance, and tuck jump.	Neuromuscular injuries	Supervised	Decision trees: J48 consolidated. Alternative decision tree. Reduces error pruning tree.	74%	Asymmetry in single leg CMJ, 75% hop distance and stick, Y-balance, plus tuck jump knee valgus and anthropometrics are the most frequent contributors to predicting injury incidences.

Rommers et al. [41]	734 U10 and U15 players during 1 season (with half of the players sustaining 1 injury)	-	Injury incidence during 1 season + Pre-season screen: anthropometric (height, weight, and sitting height) and physical fitness (strength, flexibility, speed, agility, and endurance)	Injuries from overuse or acute from pre-season results	-	Extreme gradient boosting algorithm (XGBoost)	78–85%	Proposed ML models could identify youth players with the highest injury risk.

Rossi et al. [19]	26 professionals during 1 season (931 sessions for 23 weeks)	GPS + MEMS (both from STATSports)	23 non-contact injuries were registered. + Personal features (considering previous injuries) + 12 training load variables	Injuries from overuse or acute	Supervised	Decision tree	70–100%	A previous injury can lead to another when: Training HSR > 112 m. Training HSR > 112 m and total distance monotony 3 times lower than 1.78. Training HSR > 112 m and total distance monotony 2.5 times higher than the player’s average.

Rodas et al. [15]	363 from different team sports (including soccer) from FC Barcelona during 10 leagues (55% with injury)	-	Injury attempts + Genetic markers (proteins, single nucleotide polymorphisms, genes)	Tendinopathy injuries	Supervised	Support vector machine Random forest	-	Through the analysis of genes tendinopathies can be predict. One of the most robust single nucleotide polymorphisms was rs10477683 in the fibrillin 2 gene encoding fibrillin 2.

Note = ANN: artificial neural network; MC4: BN: Bayesian Networks; HIS: hamstring strain injuries; IMU: inertial measurement unit; KNN: *K*-nearest neighbor; MC4 Decision trees;

**TABLE 4:** Stud ies that used ML to predict performance in soccer.

Ref.	Participants	Data acquisition technology	Attributes/ Features/ Variables	Aim of prediction	Machine Learning				Practical application from predicting
					ML Approach	Algorithm	Trainingalgorithm	Accuracy	
***Match/League outcomes predicting***									

Bilek and Ulas [7]	20 professional teams from English Premier League during a season (380 games)	Observation	22 situation/ performance variables: Result, match location, scoring first, cards (yelloww/red) and 16 technical variables (passes, shots, tackles, possession %)	Match outcome (win, draw, loss)	Supervised	K-means Clusters Decision trees		68–78%	To predict match outcomes, the most important variable is scoring first, and its influence depends on the quality of opposition.

Dick and Brefeld [32]	5 elite European soccer games (380 successful and 715 non-successful attacks)	Tracking system	Team in possession +Players positioning (x/y coordinates)+Game in play or halted	What game situations have a great potential to achieve successful attacks (the ball has a lower distance than 25 m from opponent team’s goal)	-	Deep reinforcement learning	Adam optimization algorithm	-	The presented deep reinforcement learning may be used to evaluate multiplayer positioning from positional data. They hypothesized that this model could be of interest to measure, together with playing speed and passes, what type of attack could predict a great success (counterattack or using cross passes).

Goes et al. [46]	26 professional teams during 4 seasons (1237 successful and 11187 unsuccessful attacks)	Semi-automatic camera system	Own GC – opponent GC Own defenders’ GC – Own midfielders’ GCThe relative phase of these variables (synchronization)	Successful attacks from collective tactical variables	Unsupervised	K-means	-		Sub-group variables (GC-GC between 2 subgroups within a team) is more sensible than team level analysis (GC-GC) to predict successful attacks. Successful attacks strongly depend on both defenders creating space for the attackers and their synchronization with midfielders’ GC.

Leser et al. [37]	20 U15 players during 279 SSG (3 vs. 2)	Camera+Trackingsystem+MEMS	30 technical/tactical parameters during two situations:1. The time instant of the shot.2. The time instant of the last action before the shot.	The efficiency of tactical patterns	Unsupervised	Clusteringtechniques	-	-	The participation of each player in each attacking pattern can be considered. As a goal in each attacking pattern was noted, the proportion of goals that is expected by each player can be identified.

Joseph et al. [23]	2 seasons, 76 matches from English 1st division Tottenham team (Premier League)	-	30 attributes.+Players, venue, and opponent quality.	Match outcomes (win, lose, or draw).Attacking force. Overall team quality. Team performance knowing own/opp. quality.	-	Expertconstructed BN Naïve BN Hugin BN MC4 KNN		From 50 to 59%	What among the selected attributes are the crucial factors affecting the outcome of a game and give some clues as to the relationships between some of those factors.

Brooks et al. [20]	20 teams from Spanish 1st division during 80% of matches from 1 season.	Camera (Opta Sports)	Pass location The time of the pass Team that made the pass Player that made the pass Intended pass recipient The origin of the pass Possessions (> 3 passes)% of passes in a possession that originate in a particular zone % of passes in a possession that have a destination in a particular zone % of passes in a possession from one zone to another zone	The success of attacks (what attacks end in a shot on goal) The players with a greater possibility to perform a shot through average pass shot value	Supervised	KNN	Cross-validation	87%	Through this model the probability can be predicted that an attack will be based on both previously recorded pass characteristics and attack success relationship, as well as which player has a greater possibility to perform a shot.

Pappalardo and Cintia [6]	6396 games and 10 million events from 6 European professional leagues (145 clubs) during 3 seasons	-	Events (pass, shot, tackle, take-on, clearance, intercept, cross, foul, aerial duel, and goalkeeping)Team and player who generated the eventEvents’ positioning Timestamp of the event	Match and league ranking outcomes	Supervised	Random forest	-	General < 80% Draws 26%	Final league ranking is related with a team typical performance (technical features).Match outcomes can be predicted with team’s performance, while predicting draws is difficult.

***Physical/physiological performance predicting***									

Bongiovanni et al. [48]	16 U15 from Italian professional club	Tools to measure anthropometric +Timing gates for yo-yo,10 and 20 m sprint, 90° change of direction test, and CMJ	16 anthropometric measures +5 created combining from 2 to 9 of these 16 initials +Physical performance from tests	Aimed to predict performance from anthropometric variables	-	Extra tree regression		High accuracy	Anthropometric features could be important to predict performance in 10/20 m sprint, yo-yo test. Upper body features (i.e., arm muscle area/circumference) could be used to predict performance in 10/20 m sprint.Lower body features right/left suprapatellar girths could influence the performance in yo-yo test. Anthropometric variables are not suitable to predict performance in change of direction and CMJ test.

Campbell et al. [5]	7918 observations after data cleaning during2013–2018 seasons	RPEand wellness surveys + GPS	8 wellness variables +iTL (RPE)+eTL (total distance and m/min)	Aimed to predict training load from wellness questionnaires	Supervised	Classification tree Regression tree Random forest		> 89%	Fluctuations in wellness status responses can lead to predicting an influence in athlete’s performance.

Geurkink et al. [34]	46 non-professional players during 2 seasons(61 training sessions)	GPS+Heart rate devices	70 iTL, eTL, personal, and supplementary variables.	Session RPE	-	GradientBoostingMachines		MAE:0.67 ± 0.09 RMSE: 0.93 ± 0.16	eTL indicators (mainly total distance covered) are the stronger RPE predictors, although including a broad range of variables, other than ELIs, may be useful.

Jaspers et al. [35]	38 professionals from 1st Netherlands division during 2 seasons.	RPE survey + GPS + MEMS	iTL indicators (RPE) + 67 eTL indicators	RPE	-	ANNLASSO	-	Lasso more precision than ANN	LASSO and ANN can predict RPE based on a large set of e TL variables.Accelerations were important eTL variables to predict RPE.

Op De Beéck et al. [40]	26 professionals during a season (pre- and in-season)	RPE survey, MEMS and GPS	Total amount not specified	Wellness	-	Gradient boosted regression tree. Naive baseline.		> 70%	A combination of eTL, iTL, and pre-season perceived wellness may indicate importance in a broad monitoring approach for performance prediction.

Perri et al. [21]	28 sub-elite players	Surveys	RPETraining time Wellness index	Match performance according to wellness index and RPE	Supervised	Regression	-	39%	ML can predict the wellness according to the load performed the day before.The ML model can be used to predict the wellness based on a targeted weekly load.

Zago et al. [43]	13 elite females in a shuttle run test	MEMS(accelerometer,gyroscopes,andbarometer)	18 features (predictors)	Turn direction Speed (before and after turn) Mechanical load during turns	Supervised	Linear regression Support vector regression Boosted decision trees ANN		> 98.4%	Although models can be extended to different angles, we showed that meaningful information on turn kinematics and energetics can be obtained from inertial units with a data-driven approach.

***Technical/tactical performance predicting***									

Chawla et al. [16]	English 1st division Arsenal team during 4 matches (2932 passes)	Video camera and human observers	Players’ trajectories +Sequence of events (touch, pass, shot, tackle, goal, etc)+Mapping of players to their respective teams (dominant regions)	Pass event	Supervised	RUSBoostclassifierMultinomiallogisticregression	-	> 90%	Considering current events, current location of all players may be possibly predict the quality of passes.

Szczepański and McHale[42]	760 matches during 2 seasons of English Premier League’s 20 teams and/or 481 players (253090 events)	Camera (Opta Sports)	Each player’s inherent player skill in a certain context.Factors that influence the probability of a pass being successful	Predict passing completion rate for the next season from the present passing completion rate.	-	Naive	-	-	This method can be used to evaluate passing skill depending on the difficulty of each attempt.

Goes et al. [17]	18 Dutch 1st division matches from 13 teams during 1 season(16943 passes from which 10481 were received)	Semi-automatic video camera (SportVU, STATS)	CentroidsPass (length, velocity) angle Convex hull SpreadMove of defensive players	Pass effectiveness	Supervised	RUSBoost classifier	-	85%	Pass success can be predicted from tactical approaches.

Link and Hoering [38]	60 matches from German Bundesliga (69,667 individual ball actions)	Semi-automatic camera system (TRACAB)	Positional data +Kick detention intervals to know: Team ball possession (a sum of individual possessions) Individual (a player) ball possession. Individual ball actions. Individual ball control.Team ball control.Team ball control without void passes.	Time that ball is in the sphere of influence of a player	-	Bayesiannetwork	K2 algorithm [12]	Ball control 97%Without ball 50%K = 0.67	Proposed model can predict how long the ball will spend in the sphere of influence of a player based on the distance between the players and the ball together with their direction of motion, speed, and the acceleration of the ball.

Montoliu et al. [39]	4 teams from Spanish 1st division League during 2 seasons	Camera	Ball possessions (attacks)Quick attacking actions (passing and moving, switching the attack, and fast break)Set pieces (direct free, indirect free, penalty, and corner kicks)	Classify ball possession, quick attacks, and set pieces (strategies)	Supervised	K-Nearest neighbor Support Vector Machine Random Forest	Back propagation [13]	67–93% Random Forest is the classifier obtaining the best classification results.	This approach can cluster team behavior into three types (ball possession, fast attack, or strategy) and to recognize the most common play patterns when playing a match.

Bialkowski et al. [22]	374 matches from 20 teams.	Semi-Automatic camera system (Prozone).Handle note.	Player location43 events (passes, shots, crosses, tackles etc.)	Team structures	Unsupervised	Expectation maximization algorithm [43] (Similar to k-means clustering).		70%. Three times better than other traditional methods (descriptive statistic, etc.)	Team formation clustering as a strong descriptor for identifying a team’s style.Assigns players to roles.It can be known when a player is in the left-wing position compared to left back.

Knauf et al. [36]	10 games. 5 from 1st and 5 from 2nd division of Bundesliga during 1 season. (31,000,000 positionings)	Camera (VIS. TRACK)	Players and ball trajectories Sequence initiation by goalkeeper Scoring opportunities (3/4 of field)	Team patterns	Unsupervised	k-medoidscluster			Through this clustering method, the player trajectories predicting how chaotic a team is or how many patterns (solution categories, quick/slow attacks with more and/or lower passes) are performed.

Note = ANN: artificial neural network; MC4: BN: Bayesian Networks; IMU: inertial measurement unit; KNN: *K*-nearest neighbor; MC4 Decision trees; LASSO: least absolute shrinkage and selection operator; RIPPER: Repeated Incremental Pruning Produce Error Reduction algorithm;

**TABLE 5:** Studies that used ML to predict talents in soccer.

Ref.	Participants	Data acquisition technology	Attributes/ Features/ Variables	Aim of prediction	Machine Learning			Practical application from predicting
					ML Approach	Algorithm	Accuracy	
***Talent identification***								

Barron et al. [8]	966 players (low level: 209; Football League Championships: 637; English premier league: 120)	ProZone’ s MatchViewer system	347 technical indicators: total number, accuracy, and consistency of passes, tackles, possessions regained, clearances, and shots.	Using data set from previous seasons, the aim was to know the level of a player, comparing where he played in the following seasons after data recording.	-	ANN	61–79%	From data set recorded in the previous seasons, proposed ML could predict where the player will play in the following seasons (in lower leagues, championship, or English Premier League). ANN can accurately predict the career trajectory of a player.

Sheng et al. [10]	13^th^ Chinese national games data as well as the training matches data of the Shanghai U20 soccer team	Tracking camera	Technical actions (iceberg figure) + Players’ positioning (heat map)	Select most potential players	-	GreenSea broad learning system	-	Using this model, game analysis could help coaches in decision-making regarding players with the most potential and their improvement.

***Player transferences***								

Ćwiklinski et al. [9]	4700 players from 156 clubs belonging to the eight most popular leagues	Notation	29 technical variables + 7 psychological variables + 4 outcome variables (matches played, won, etc.)	The aim was to predict if a player transferal between two teams is successful (successful = player performance was (i) up from the previous season before transference and (ii) above the mean of his new team.	-	Random forest Naive Bayes AdaBoost	-	A model can predict if a player will be a successful transfer based on some technical, psychological, and physical (outcome) variables.

***Identification of lineups issues***								

García-Aliaga et al. [33]	Matches over 7 seasons from 18 national leagues. A total of 50,000 matches and 30,000 players.	Tracking camera (OPTA)	Individual technical-tactical features	Players’ playing position based on technical/ tactical features	Supervised	RIPPER	> 73%	The ML has the potential to predict a player’s most suitable playing position based on his/her technical-tactical performance.

Sheng et al. [10]	13^th^ Chinese national games data as well as the training matches data from the Shanghai U20 soccer team	Tracking camera	Technical actions (iceberg figure) + Players’ positioning (heat map)	Analyze games and make team tactics	-	GreenSea broad learning system	-	Using this model, game analysis could help coaches in decision-making regarding the team tactics.

Note = ANN: artificial neural network; MC4: BN: Bayesian Networks; IMU: inertial measurement unit; KNN: *K*-nearest neighbor; MC4 Decision trees;

## DISCUSSION

The present systematic review was to systematically identify original studies that applied ML with soccer data, highlighting current possibilities in ML and future applications. The main findings were: (1) the best attempts at predicting injury incidences are derived from pre-season screening, training load, genetics, or risk factor surveys (personal, psychological, and neuromuscular), that through algorithms such as decision trees can predict injury risk at an accuracy greater than 66%; (2) performance predictive ML models were used in three ways: predicting outcomes, physical/physiological performance, and technical/tactical performance. (2.1) notational analysis and positional data can lead to predicting through ML the match outcomes or even the final ranking of a league. (2.2) ML approaches can help to identify the best contributors and determinants to explain wellness and training load magnitudes and that eventually can give some important orientations to coaches. (2.3) technical and tactical inputs can reveal the passing effectiveness and classify team styles through the ML algorithms. (3) Finally, ML algorithms can lead to predicting a young talent, predicting a player’s successful transferal between teams, and the most suitable configuration of starter players for a competition, mainly using technical and positional data as input.

In soccer, the laws establish a framework of action that, in a certain way, guides players performance beforehand [44]. However, soccer game dynamic, where two teams play against with the same objective but on opposite sides, allows players to perform a wide range of actions, only allowing for the possibility of hypothesizing about what is going to happen [45]. In this sense, coaches are involved in a decision-making game, in which actions are taken that, despite being based on tracking objective data, necessarily have to be intuitive due to the unpredictability of events. However, technological development has allowed the arrival of prediction algorithms (e.g., ML), which has made use of the large amount of data recorded and storage available, becoming them as input, and making “learning” processes possible in order to base outputs into a large amount of data stored, allowing the possibility of what is going to happen to be anticipated in three aspects: (1) injury prevention, (2) performance, and (3) talent identification.

### Injury prevention predicting

Due to the expensive process of recovery and rehabilitation for players, as well as the seasonal costs of medical care, injury involves a great economic cost to a soccer club. For example, Rossi et al. [19] estimated a minimum of 11,583 euros/player is spent during a season (931 sessions performed during 23 weeks) by a professional soccer club. In this sense, the newly developed potential to anticipate the future has encouraged sport scientists to use ML prediction models to try and help coaches in decision-making and training design, with the aim of reducing the number of severe muscle injuries [2, 3, 18, 41] and of highlighting the most predictive features in this field [4, 15, 19].

The proposed ML algorithms were mainly based on decision trees (ADTress [2], XGBoost based on trees classifiers [18, 41], C4.5., SimpleCart, ADTree, and Random Tree [3], and J48 consolidated, reduced error punning tree, and alternative decision trees [4]), although Support Vector Machine and Random Forest were also considered for injury predicting [15]. The inputs were based on injury incidences and personal, psychological, and neuromuscular risk factors [2, 3]; pre-season anthropometrics (height, weight, and sitting height) and physical fitness (strength, flexibility, speed, agility, and endurance) screening [4]; and training load (distance covered at different intensities (total distance, high speed running (HSR) and explosive distance), metabolic load related features, accelerations/decelerations, dynamic stress, and fatigue index) [19]. For example, Ayala et al. [2] through a supervised ADTree and personal, psychological, and neuromuscular features predict more than 78% of strain injury occurrences, while Rommers et al. [41] proposed a model which predicted, with 78–85% of predictive attempts, players that had the highest injury risk. All these authors, like López-Valenciano et al. [3] making possible the application of ML to injury risk management strategies in order to reduce muscle injury incidence, or even, to predict changes in a certain part of players’ body such as DiCesare et al. [18], who proposed that through XGBoost based on trees classifier, with more than 83% of prediction attempts, it can be predicted the longitudinal changes in the brain caused by head impacts. These ideas may only lead to the highlighting of ML as an option to reduce injury incidences (following these proposals with a greater accuracy than 66% of prediction attempts), offering crucial information that may be used for training design strategies during the certain moments or with certain players that have a greater likelihood of suffering injuries.

Furthermore, some authors have tried to point out more specific indications, highlighting the most frequent contributors to the prediction of injury incidences [4, 15] or practical applications [19]. Specifically, Rodas et al. [15] analyzed 363 players from different team sports (including soccer) from FC Barcelona (Spain), and highlighted that using data about injury incidences and genetic markers (proteins, single nucleotide polymorphisms, genes) may predict tendinopathy injuries. Specifically, the authors found that one of the most robust single nucleotide polymorphisms to predict tendinopathies was rs10477683 in the fibrillin 2 gene encoding fibrillin 2, a component of connective tissue microfibrils involved in elastic fiber assembly [15]. However, due to the impossibility of using these inputs for most teams – mainly non-professional teams – the use of pre-season screening may be a more achievable strategy. In fact, Oliver et al. [4] have highlighted that a single leg counter movement jump, 75% hop distance and stick, Y-balance, plus tuck jump knee valgus, and anthropometric features are the major predictors for injury incidences; at least with young soccer players. Beyond, and not only considering a prediction test in the initial stages of a season, Rossi et al. [19] emphasized some conditions that through popular and widely used tracking systems may be considered. Specially, the authors highlighted that a previous injury can lead to another when: (1) training HSR is > 112 per/min; (2) training HSR is > 112 m/min and total distance monotony is three times lower than 1.78; and (3) training HSR is > 112 m/min and total distance monotony is 2.5 times higher than the player’s average. Therefore, these practical applications may be used by clubs to predict injury incidences and their negative consequences.

In summary, data about injury incidences, together with other additional variables such as pre-season screening, training load, genetics, or risk factor surveys (personal, psychological, and neuromuscular), may be used as inputs to predict injury incidences through ML algorithms such as decision trees, which have been the most used algorithms for injury prevention. Practically, performing a pre-season test, or even if possible the use of tracking systems and the analysis of genetic markers, may be useful to predict players or situations with a higher percentage of injury risk.

### Performance predicting Match/League outcomes predicting

Among the seven included articles [6, 7, 20, 23, 32, 37, 46] focusedin the use of ML in match performance, five of them were able to produce an accurate prediction regarding the match performance [6, 7, 20, 23, 46]. The studies of Bilek and Ulas [7], Joseph et al. [23], and Pappalardo and Cintia [6] made prediction attempts regarding the match outcome, while the studies of Goes et al. [46] and Brooks et al. [20] attempted to predict the successful attacks. Without a prediction attempt, the studies of Dick and Brefeld [32] and Leser et al. [37] focused on detecting the situations with a high potential of success and on analyzing the efficiency of tactical patterns, respectively.

The approach used by the studies conducted to predict the match outcome were different; namely considering the usable variables [6, 7, 23]. While the studies of Bilek and Ulas [7] and Pappalardo and Cinti [6] have focused on regular notational analysis events (e.g., passes, shots, possession), the study of Joseph et al. [23] was centered on the presence of specific players, the quality of the opposing team or the venue. Using the k-means, clusters and decision trees, the study of Bilek and Ulas [7] using 22 situational variables from 380 games and 760 observations in the English Premier League was able to predict match outcomes with between 68 and 78%, in which it was found that situational variables and match results were determinant. Interestingly, it was revealed that scoring first and the quality of opposition was determinant [7]. In another study considering 6000 games and 10 million events in six European leagues, it was found that victory and defeat can also be explained with team’s performance, and that final league position is also dependent on technical performance [6]. Interestingly, both studies [6, 7] have revealed a clear link between match outcomes and technical performance.

Looking for classification of attacking situations, and using classical notational events (e.g., pass, possession, pass location), the study conducted by Brooks et al. [20] on Spanish teams allowed passes to be classified in heat maps, and from those the patterns of the teams to be detected and the KNN model to be run. The study concluded that the locations of the origins and destinations of passes in a possession are correlate highly to whether that possession will end in a shot [20]. However, technical performance measures are just another product or outcome, which may not be the best information to adjust the training process [47]. Measures related to processes identifying which behaviors can be conducive to better efficacy and ultimately to performance should be also considered. Thus, considering the use of the bi-dimensional analysis of player relationships, the study of Goes et al. [46] classified successful attacks by taking into consideration the relationship between the geometrical centers of both teams and the synchronization level. Using a sample of 11187 attacks (successful and unsuccessful), from the results it was possible to identify that successful attacks were related to decreases in the synchrony of intra-team and inter-team subgroup analysis, thus with the creation of space [46].

Using a mixed approach between classical notational measures and information related to player positioning in the Cartesian space, the study of Dick and Brefeld [32] revealed that the implemented model was able to evaluate multiplayer positioning based on positional data and that attacking can be predicted for its efficacy by adding information about playing speed and passes. Additionally, the study of Leser et al. [37] allowed the identification that offense attempts have the highest scoring probability when played exploring the wings near to the goal lines and when the final pass, before the shot, is given against the direction of play.

In brief, the included studies about predicting match outcomes had focused on two main aspects: predicting the match outcome by using classical notational variables and identifying the efficacy of attacks by considering notational analysis events and positioning data from the players. The studies considering the efficacy of actions were focused on attack [6, 7, 20, 32, 37]. Future studies should focus on defensive processes, namely focusing on considering behaviors that are conducive to success and not only on events (product measures).

### Physical/physiological performance predicting

From the included articles in this section, one study focused on determining the capacity of anthropometry to influence physical performance [48], three studies related wellness data with training load and match performance [5, 21, 40], two studies tested the prediction of rate of perceived exertion (RPE) based on training load measures [34, 46] and one focused on using inertial units to identify the kinematics and energetics of player demands [43].

Regarding the use of extra tree regression for determining the contribution of soccer players’ anthropometric features to predict their physical performance, it was possible to ascertain the determinacy of these features for sprinting and aerobic fitness [48]. Despite the small sample used, it was also possible to conclude that machine learning can be used to identify the importance of a specific measure to predict a player’s performance [48]. Naturally, more studies should be conducted, and with greater samples. Eventually, comparison between prediction methods should also be considered.

Regarding the studies focusing on wellbeing parameters and the relationship with training load and match performance, the study conducted by Campbell et al. [5] revealed a limited predictive capacity of wellness to determine internal and external load, even considering different approaches (regression, classification and random forest models). The findings also allowed the suggestion that new wellness questionnaires should be considered to test the sensitivity to training load or vice versa, since a previous systematic review also confirmed that wellness and training load are minimally to slightly related [49]. Changing the approach, a study conducted on 26 professional players tried to predict perceived wellness based on preceding load and perceived wellness using individual machine learning models [40]. The analysis revealed that the combination of training load parameters with preceding perceived wellness resulted in the best predictive performance and that using cumulative load did not improve the predictions. This study also suggests implementing wellness questionnaires as part of a well-implemented monitoring strategy [40]. Also testing the influence of match load on wellness, a study conducted on sub-elite players [21] revealed the importance of match load in wellness responses, thus confirming the relevance of impact on fatigue. Therefore, among these three wellness-related articles [5, 21, 40] it is possible to argue for the sensitivity of wellness to training and match load, but that there is a lack of relevance on the demands and training load.

Testing the relationships between different training load measures, two studies have used machine learning to predict the RPE [34, 35]. In a study conducted over two seasons using a sample of 61 training sessions [34], it was possible to identify that from different external load measures, distance covered was the best predictor of RPE, despite internal load measures (heart rate zones, training impulse) being the strongest predictors of the session-RPE [34]. Also using machine learning to determine relationships between external and internal load measures, it was possible to find that RPE was predicted by both internal and external load measures, namely considering high-magnitude decelerations [35]. Therefore, it seems that session-RPE (a perceptive scale) is highly capable of connecting information and dependency from objective measurements from internal and external load.

In summary, it is possible to conclude that ML approaches can help to identify the best contributors and determinants to explain wellness and training load magnitudes, and this eventually can help in the future to reduce the amount of data collected and improve the efficacy of the monitoring process in players. Additionally, it can be expected that ML may help to determine the best predictors of athletic performance, thus giving some important orientations to coaches.

### Technical/tactical performance predicting

Among the included studies, three of them were focused on identifying the effectiveness of passes [16, 17, 42], two on identifying ball possession and circulation [39, 40], and two on classifying the team’s patterns of play [22, 36]. Five of the studies tested predictions [16, 17, 22, 38, 39]. Regarding the studies that proposed to identify the effectiveness of passes, one of them [16] proposed a model able to learn a classifier to rate the quality of passes with an accuracy of 85%, revealing that machine learning had similar efficacy to a human observer. With a different aim, the study of Szczepański and McHale [42] tested the capacity to estimate each player’s passing skill and make predictions for the next season. The model implemented in that study revealed a good capacity to predict the next season’s completion rate based on the past, and that variations in the difficulty of passes attempted in both seasons had a determinant impact in the observed performance of some players [42]. Not using observational actions but using information from position data and geometrical interactions between players, and considering 16943 passes [17], it was possible to classify how well a pass disrupts the opposing defense. Despite the advances in this field of detecting passes, interaction between different kinds of measures should be considered; namely integrating data from events and position data about collective dynamics that lead to pass success.

Analysis of process and outcomes is necessary, mainly to explain how teams behave. Considering positional data and team ball possession, a study conducted in the German Bundesliga [38] described models for detecting individual and team ball possession. The model proposed was able to determine individual possession and consider the time spent in different regions of the pitch based on distance between players and their direction of motion, speed and the acceleration of the ball [38]. Interestingly, it was found by the authors that individual ball possession influenced passes, tackles and shots on goal [38]. In another study also focusing on ball possession [39], it was found that information about number of ball possession, attacking actions and set pieces (direct free, indirect free, penalty, and corner kick) was able to classify the team’s behavior in three categories: ball possession (low pace), fast attack or strategy. Among the models tested, the random forest was the one allowing the best classification result, even considering comparisons with neural network-based methods [38].

Also considering the classification of team behavior, a study conducted over 374 matches [22] analyzed player tracking data to describe the formation of the team. Using an expectation maximization algorithm it was possible to assign players to a role and classify the team’s formation [22]. In a different approach, a study conducted over 10 matches [36] used the k-medoids cluster to analyze the chaotic level of the teams and the different patterns emerging from the match considering player trajectories.

In summary, the research using machine learning for technical/tactical performance reveals the capacity of models to predict the passing effectiveness and to classify team styles. This can be interesting for a future automatic analysis about the behaviors of the teams and for exporting interpretations for coaches to adjust the training drills to the needs of the teams.

### Talent identification

The current trend focusing on talent has defined which term as a dynamically varying relationship molded by the constraints imposed by the physical and social environments, the task experienced, and the personal resources of a player [50], highlighting talent identification in one of the more important challenges in soccer, not only during early ages [8, 10], but also for player transferal between soccer clubs [9]. In addition, ML can be used in this way to predict the most suitable players for decision-making for competition starter players (choosing players and playing positions) [10, 33].

To date, it has been systematically reviewed that technical and time-motion variables are clustered into principal components that explain the behavior of soccer players [51]. Based on these ideas, the most used inputs (features) to identify the talent of soccer players are technical variables such as passes, tackles, possessions, clearances, or shots [8–10], as well as variables extracted from tracking systems [10], and psychological indicators [9]. For example, Barron et al. [8] analyzed 966 soccer players (209 from low level, 637 for Championship Football League, and 120 from English Premier League), and with an accuracy of between 61 and 79% of attempts, the authors predicting players’ career trajectory using 347 technical attributes. Similarly, Sheng et al. [10] assessed a ML algorithm (Green-Sea broad learning system) highlighting the potential of the model to predict the players with the most potential and their improvement. However, talent identification is not only supposedly relevant with young soccer players, but also for professional players. For example, Real Madrid, based on seven successful seasons that Eden Hazard performed, contracted the player with one of the most expensive transfers involving an English club in history, resulting in a non-successful transfer [9]. So, in this way, Ćwiklinski et al. [9] enrolled 4700 players from 156 clubs from one of the eight major leagues, and using technical (n = 29), psychological (n = 7), and match outcome (n = 4) features, they predict successful and non-successful player transferals, understanding success such as those transferals where the player’s performance was up from the previous season and above the mean of his new team [9]. Lastly, and in agreement with the use of technical features, Sheng et al. [10] and García-Aliaga et al. [33] highlighted the potential use of ML to predict the most suitable playing position for a player [33], as well as predicting more suitable team tactics which may help coaches in decision-making [10] using both technical and positional features. So, technical features seem to be the most suitable inputs to predict talents in both young and professional soccer; however, and despite the large number of players involved in these studies, future studies are needed to highlight what are the most frequent contributors to predicting.

### Study limits

Due to the unpredictable nature of soccer, the indications coming from ML should be considered with caution, taking into account that they may be only one piece of advice that can help coaches in decision-making in different ways.

Comparison of studies in this area is complicated nowadays for the following reasons:

1) Most of the selected studies use datasets created by the researchers themselves, which are not publicly accessible. This makes the published research not reproducible. Then, since ML models depend upon the quality and amount of dataset, authors may include them, at least, as a separate file in future research.
2) No studies have analyzed the amount of data input needed make to a relevant predictive attempt which makes accurate predicting available, further studies should analyze this issue.
3) Different data sources have been used in each article: subjective variables, biomechanical measurements, images or videos, mainly.

In any case, of all the articles selected in this review, and independently of the objective pursued, only 4 have used unsupervised algorithms [17-22-36-37], which indicates that better results are obtained for the applications analyzed with supervised algorithms.

## CONCLUSIONS

Due to the development of technology, a large amount of data has become a widely used tool to manage training and competitions, using them to make decisions. Today, the development of algorithms capable of learning as more data are collected and stored has become known as ML in an important topic in soccer. Practically, the application of ML to soccer has been performed using a wide range of predictive algorithms, where those most considered are decision trees. Overall, three types of predictions can be made: (1) injury, (2) performance, and (3) talent predicting. (1) For injury prevention, pre-season screening, training load, genetics, or risk factor surveys (personal, psychological, and neuromuscular) can be used as inputs in a ML algorithm, where it may learn from the large amount of data added. (2) Performance predictive ML models were used in three ways: predicting outcomes, physical/physiological performance, and technical/tactical performance. While notational analysis and positional data can lead to predict match outcomes or even the final ranking of a league through ML, its approaches can help to identify the best contributors and determinants for explaining wellness and training load, and technical and tactical inputs can reveal passing effectiveness and classify team styles. (3) Finally, ML algorithms can lead to predicting a young talent, to predict a successful player’s transferal between teams, and the player lineup most suitable for a competition, mainly using technical and positional data as inputs. Therefore, the use of ML may help team staff members in decision-making to predict dose-response relationships, reducing the chaotic nature of this team sport.

Publishing the datasets developed the authors could be a very good way of contributing to solving this lack of publicly available data and design comparative studies in the future.

## Funding

MRG gratefully acknowledge the support of a Spanish government subproject Integration ways between qualitative and quantitative data, multiple case development, and synthesis review as main axis for an innovative future in physical activity and sports research [PGC2018-098742-B-C31] (Ministerio de Ciencia, Innovación y Universidades, Programa Estatal de Generación de Conocimiento y Fortalecimiento Científico y Tecnológico del Sistema I + D + i), that is part of the coordinated project New approach of research in physical activity and sport from mixed methods perspective (NARPAS_MM) [SPGC201800X098742CV0]. FMC: This work is funded by Fundação para a Ciência e Tecnologia/ Ministério da Ciência, Tecnologia e Ensino Superior through national funds and when applicable co-funded EU funds under the project UIDB/50008/2020. No other specific sources of funding were used to assist in the preparation of this article.

## Conflicts of interest/Competing interests

The authors declare that they have no conflicts of interest relevant to the content of this systematic review.
